# A Comparative Prospective Study to Assess Efficacy of Intralesional MMR (Measles, Mumps, Rubella) Vaccine and Intralesional Vitamin D3 in Treatment of Nongenital Warts

**DOI:** 10.1002/hsr2.70782

**Published:** 2025-05-05

**Authors:** Bibisha Baaniya, Suchana Marahatta, Raunak Dahal, Nidhi Shah

**Affiliations:** ^1^ B.P. Koirala Institute of Health Sciences Dharan Nepal

**Keywords:** immunotherapy, measles, mumps and rubella vaccine, Vitamin D3, warts

## Abstract

**Background and Aims:**

Intralesional immunotherapy is an upcoming method of wart treatment with fewer side effects and this study aims to assess and compare the efficacy of intralesional MMR vaccine and vitamin D3 in the treatment of nongenital warts.

**Methods:**

A comparative prospective study was done with a total of 61 patients divided into two groups. Thirty‐one patients were treated with intralesional MMR, while 30 patients received intralesional vitamin D3. In both groups, a maximum of five sessions were carried out every 3 weeks and follow‐up was done for 3 months. *χ* ^2^, Fisher's exact, and multivariate analyses assessed variable relationships.

**Results:**

In the MMR group, 80.6% of patients achieved complete response, 6.5% had excellent response, 9.7% showed good response, and 3.2% had no response. Similarly, in the vitamin D3 group, 73.4% achieved complete response, 20.0% had excellent response, 3.3% showed good response and 3.3% had no response. The difference in response rates between the two groups was statistically insignificant. One patient (4.0%) in the MMR group and three patients (13.6%) in the vitamin D3 group experienced recurrence within 3 months of follow‐up, but this difference was not statistically significant. Both treatments were well tolerated. Pain was universal, while swelling (60%), hyperpigmentation (30%), and pruritus (16.7%) were more common with vitamin D3. In logistic regression analysis, pain duration was significantly higher in the Vitamin D group compared to the MMR group (*p* < 0.001). Fever (MMR) and hypervitaminosis D3‐related fatigue (vitamin D3) were rare. No severe adverse events occurred.

**Conclusions:**

Both intralesional MMR and vitamin D3 showed positive response and were well tolerated and comfortable modalities for the treatment of warts. However, recurrence rate and side effects were higher with Vitamin D3 than MMR.

Clinicaltrials.gov: NCT04428359.

## Summary

- Intralesional antigen immunotherapy is a safe and effective therapeutic modality that warrants more clinical trials to further evaluate its effectiveness.
- Measles, mumps, rubella vaccine and vitamin D showed positive response and were well tolerated in the treatment of warts but side effects were higher with Vitamin D3.
- Both Measles, mumps, rubella vaccine and vitamin D3 via intralesional injection can be used to treat nongenital warts.

Warts are papulonodular lesions of the epidermis caused by the human papillomavirus (HPV) [1]. Cutaneous warts affect 7% to 10% of the general population, with peak occurrence between the ages of 12 and 16 years [2].

HPV comprises over 100 subtypes. HPV infections are currently classified into the following categories:

1. Nongenital (Cutaneous)
2. Mucosal or Anogenital
3. Epidermodysplasia Verruciformis

HPV subtypes 6 and 11 are considered low‐risk and are typically associated with the development of genital warts and low‐grade precancerous lesions. In contrast, subtypes 16 and 18 are high‐risk and are responsible for high‐grade intraepithelial lesions that can progress to malignancies [3].

Destructive therapies are often uncomfortable, require multiple sessions to treat each wart individually, and have inconsistent efficacy. They are also associated with a high rate of recurrence and potential scarring [4]. As a result, immunotherapies such as imiquimod, cimetidine, levamisole, zinc sulfate, tuberculin, MMR, candidin, trichophyton, BCG, and vitamin D are gaining popularity, especially for treating refractory cutaneous and genital warts [5].

The precise mechanism of immunotherapy remains unclear, but it is thought that injecting the HPV‐infected tissue triggers a delayed‐type hypersensitivity reaction, leading to the elimination of HPV‐infected cells [6]. Intralesional administration of the MMR vaccine has shown promise in the treatment of warts by stimulating a Th1‐type immune response. This immune activation promotes the release of key cytokines such as TNF‐α, IL‐2, IL‐4, IL‐5, and IFN‐γ, leading to a delayed‐type hypersensitivity reaction that targets both the viral components of the vaccine and HPV‐infected cells [7]. Conversely, vitamin D3, when injected intralesionally, is thought to activate toll‐like receptors on macrophages, enhancing the expression of vitamin D receptor (VDR) and 1α‐hydroxylase. This upregulation supports the production of antimicrobial peptides, which may contribute to the immune‐mediated resolution of wart lesions [7, 8, 9]. Additionally, the trauma caused by the injection may contribute to the resolution of warts in previously sensitized individuals [10]. The inclusion of three different antigens in the MMR vaccine enhances sensitivity to the injected antigen and reduces the risk of anergy [11]. However, to determine the ideal candidate for immunotherapy, particularly with the MMR vaccine, several factors may need consideration, including the disease's active state, its duration, history of sensitization, and the use of broad‐spectrum antibiotics, to achieve the best possible outcome [12].

The intralesional MMR vaccine has been in use longer than intralesional vitamin D3, with more comparative studies demonstrating its efficacy [13]. On the other hand, the advantages of vitamin D3 over the MMR vaccine include its cost‐effectiveness, no need for cold chain maintenance, easy availability, and suitability for use in immunosuppressed patients.

## 1 Materials and Methodology

Our study was approved by Institutional Review Committee (IRC), BPKIHS, (#IRC/1607/019) and Nepal Health Research Council (NHRC) (#858‐2019) and registered in Clinicaltrials.gov (NCT04428359). It was a comparative prospective study conducted at the Dermatology outpatient department of BPKIHS, Dharan from June 2020 to January 2021. The study uses a 95% confidence interval and 80% power to estimate sample size. Literature review indicates that intralesional MMR showed an 84.6% improvement, while intralesional vitamin D showed a 40% improvement [14, 15].

Now using the sample size estimation formula for 2 proportion

$$n=\frac{2p\left(1‐p\right)\;{\left(Z\beta +Z\alpha /2\right)}^{2}}{\left(p1‐p2\right)}$$

*n *= sample size for each group

*p*_1_ = 0.846

*p*_2_ = 0.40

*p *= (*p*_1_ + *p*_2_)/2

*Z*_*α*/2_ = 1.96Z_*β*_ = 0.842Using above formula, *n* = 18.54

Considering the loss to follow‐up, the final sample size for each group = 30.

Sixty‐six patients, aged 12 years and older, with three or more nongenital warts or a single wart in difficult‐to‐treat areas (such as periungual, palms, and soles) were enrolled in the study. Genital warts were excluded as ulceration has been recorded with IL Vitamin D [16, 17].

Exclusion criteria included any wart treatment within the last 4 weeks, a history of allergic reactions, acute febrile illness, immunosuppression, pregnancy, lactation, a history of asthma, convulsions, keloidal tendency, refusal to consent, and signs of hypervitaminosis D.

After obtaining written informed consent, patients were randomly assigned to either the MMR group or the Vitamin D3 group using a computer‐generated random number table and only the participants were blinded. A preset proforma was completed for each patient. In the Vitamin D3 group, serum vitamin D levels were monitored after the 3rd or 5th dose. Photographs of the lesions were taken before the first treatment session, at every treatment session, and 3 months after the final session.

Freeze‐dried MMR vaccine, stored at 2°C–8°C, was reconstituted with 0.5 mL of distilled water and injected immediately. Patients in the Vitamin D3 group received 0.5 mL of Inj. Vitamin D3 (600,000 IU/mL) in each session, following an injection of intralesional lignocaine. In both groups, up to 5 warts were injected at a time using a 31 G insulin syringe. Treatments were repeated at 3‐week intervals for a maximum of five sessions or until complete resolution of the warts, whichever occurred first [9, 18].

The clinical improvement was rated by the patient and physician global assessment using a visual analog scale score at each visit (Table 1) [19]. Immediate and late adverse effects were assessed after each treatment session, and necessary investigations and interventions were performed as needed. Following the final treatment, patients were monitored monthly for 3 months to check for any recurrence. The status at the end of this follow‐up period was considered the endpoint of the study.

**Table 1:** Patient and physician global assessment using visual analog scale (VAS) score and comparison of response rates with IL MMR and vitamin D3 (MMR = Measles, Mumps, Rubella vaccine, *N* = number of participants).

Grades of clinical improvement	Definition	VAS score	Intention to treat analysis		Test of Significance	Per protocol analysis		Test of Significance
			MMR group (31) *N* (%)	Vitamin D3 group (30) *N* (%)		MMR group (30) *N* (%)	Vitamin D3 group (26) N (%)	
Complete response	Complete disappearance of warts including distant ones and skin texture at the site is restored to normal	100%	25 (80.6)	22 (73.4)	*χ* ^2^ = 0.461 *p*‐value = 0.50	25 (83.3)	20 (76.9)	*χ* ^2^ = 0.363 *p*‐value = 0.55
Excellent response	Reduction in size and number including distant ones and few residual warts still visible	75%–99%	2 (6.5)	6 (20.0)		2 (6.7)	4 (15.4)	
Good response	Some reduction in size only including that of distant ones but no decrease in the number of warts	50%–74%	3 (9.7)	1 (3.3)		2 (6.7)	1 (3.8)	
Poor or no response	No significant change in size and the number of warts	0%–49%	1 (3.2)	1 (3.3)		1 (3.3)	1 (3.8)	

### 1.1 Statistical Analysis

Data processing was performed using Statistical Package for the Social Sciences version 16.0 (SPSS Inc., Chicago, III., USA). Descriptive statistics were used for the mean, proportion, and standard deviation. In inferential analysis, significance tests like chi‐square, Fisher's exact test were used to find the association between groups. A *p*‐value of< 0.05 was considered to indicate statistical significance at a level of significance of 5% and the test was two‐sided. Variables with a *p*‐value ≤ 0.01 in the bivariate analysis were included in multivariate analysis (Regression analysis).

## 2 Results

**Figure 1:**
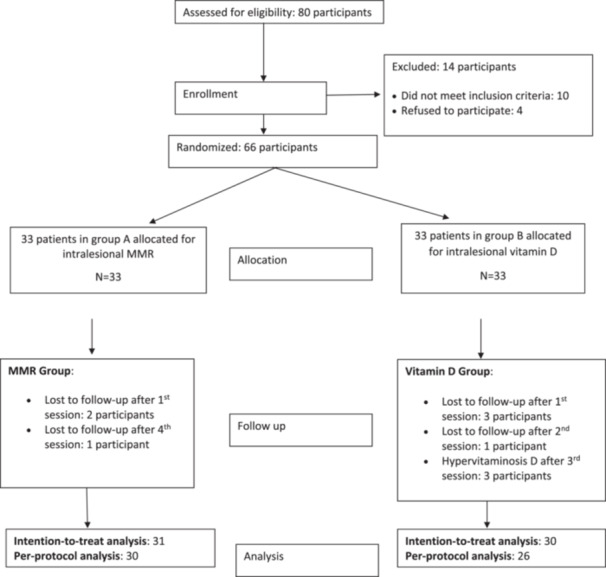
CONSORT Flow Diagram: A flowchart illustrating participant inclusion, randomization, and progression throughout the study.

A total of 66 patients were enrolled in the study, with 33 in each group. Two patients in the MMR group and three in the Vitamin D3 group were lost to follow‐up after the first session and were therefore excluded from the analysis. This resulted in 31 patients in the MMR group and 30 patients in the Vitamin D3 group. In the MMR group, one patient was lost to follow‐up after receiving 4 doses, while in the Vitamin D3 group, one patient was lost to follow‐up after the 2nd dose, and three others did not receive further treatment due to hypervitaminosis D after the 3rd dose. Their responses were recorded and included in the intention‐to‐treat analysis but excluded from the per‐protocol analysis. (Figure 1) All demographic and clinical data for both groups were comparable, with no statistically significant differences (Table 2)

**Table 2:** Comparison of demographic and clinical data of MMR group and Vitamin D3 group (MMR = Measles, Mumps, Rubella vaccine, *N* = number of participants, OR = odds ratio, CI = confidence interval, R = reference group, cm = centimeter, *significant *p*‐value < 0.05).

Characteristics	Category	Response			*p*‐value	
		MMR group (31) *N* (%)	Vitamin D3 group (30) *N* (%)	Test of significance		OR (95% CI)
*Socio–demographic profile*						
Age	< 20	15 (48.4)	11 (36.7)	*χ* ^2^ = 1.847	0.92 0.62 0.34	1.10 (0.15–7.74)
	20–30	10 (32.3)	11 (36.7)			1.65 (0.22–11.99)
	30–40	3 (9.7)	6 (20.0)			3 (0.31–28.84)
	≥ 40	3 (9.7)	2 (6.7)			R
Gender	Female	16 (51.6)	14 (46.6)	*χ* ^2^ = 0.149	0.70	1.21 (0.44–3.33)
	Male	15 (48.4)	16 (53.4)			R
Ethnicity	Brahmin/Chhetri	9 (29.0)	9 (30.0)	*χ* ^2^ = 1.770	> 0.99 0.48 0.81	1 (0.11–8.7)
	Madhesi	2 (6.5)	5 (16.7)			2.5(0.19–32.19)
	Janajati	18 (58.1)	14 (46.7)			0.77 (0.09–6.23)
	Others	2 (6.4)	2 (6.6)			R
Address	Sunsari	22 (71.0)	19 (63.3)	*χ* ^2^ = 0.403	0.53	1.41 (0.48–4.14)
	Outside Sunsari	9 (29.0)	11 (36.7)			R
*Clinical characteristics*						
Type of Wart	Palmoplantar	22 (53.7)	19 (46.3)	*χ* ^2^ = 1.351	0.53 0.95 0.68	0.70 (0.24–2.06)
	Periungal	6 (50)	6 (50)			1.04 (0.29–3.68)
	Verruca vulgaris	17 (48.6)	18 (51.4)			1.23 (0.44–3.41)
Progression	Gradual	23 (74.2)	27 (90.0)	*χ* ^2^ = 2.577	0.11	0.31(0.07–1.34)
	Rapid	8 (25.8)	3 (10.0)			R
Past treatment	None	21 (67.7)	19 (63.4)	*χ* ^2^ = 0.131	0.72	
	Traditional	2 (6.5)	1 (3.3)			1.21 (0.42–3.50)
	Medical	8 (25.8)	10 (33.3)			
Duration of illness	0–6	6 (19.4)	4 (13.3)	*χ* ^2^ = 0.800	0.41 0.18	0.56 (0.13–2.25)
	6–12	4 (12.9)	1 (3.3)			0.21 (0.02–2.02)
	≥ 12	21 (67.7)	25 (83.4)			R
Number of lesions	0–10	8 (25.8)	15 (50.0)	*χ* ^2^ = 4.745	0.96 0.35 0.42	0.93 (0.07–11.99)
	10–50	20 (64.5)	12 (40.0)			0.30 (0.02‐3.67)
	50–100	2 (6.5)	1 (3.3)			0.25 (0.008–7.45)
	≥ 100	1 (3.2)	2 (6.7)			R
Size of largest wart (cm)	0.2–1	14 (45.2)	12 (40.0)	*χ* ^2^ = 4.686	0.22 0.03	2.57 (0.56–11.72)
	1–2	8 (25.8)	15 (50.0)			5.62 (1.17–26.85)
	≥ 2	9 (29.0)	3 (10.0)			R

**Figure 2:**
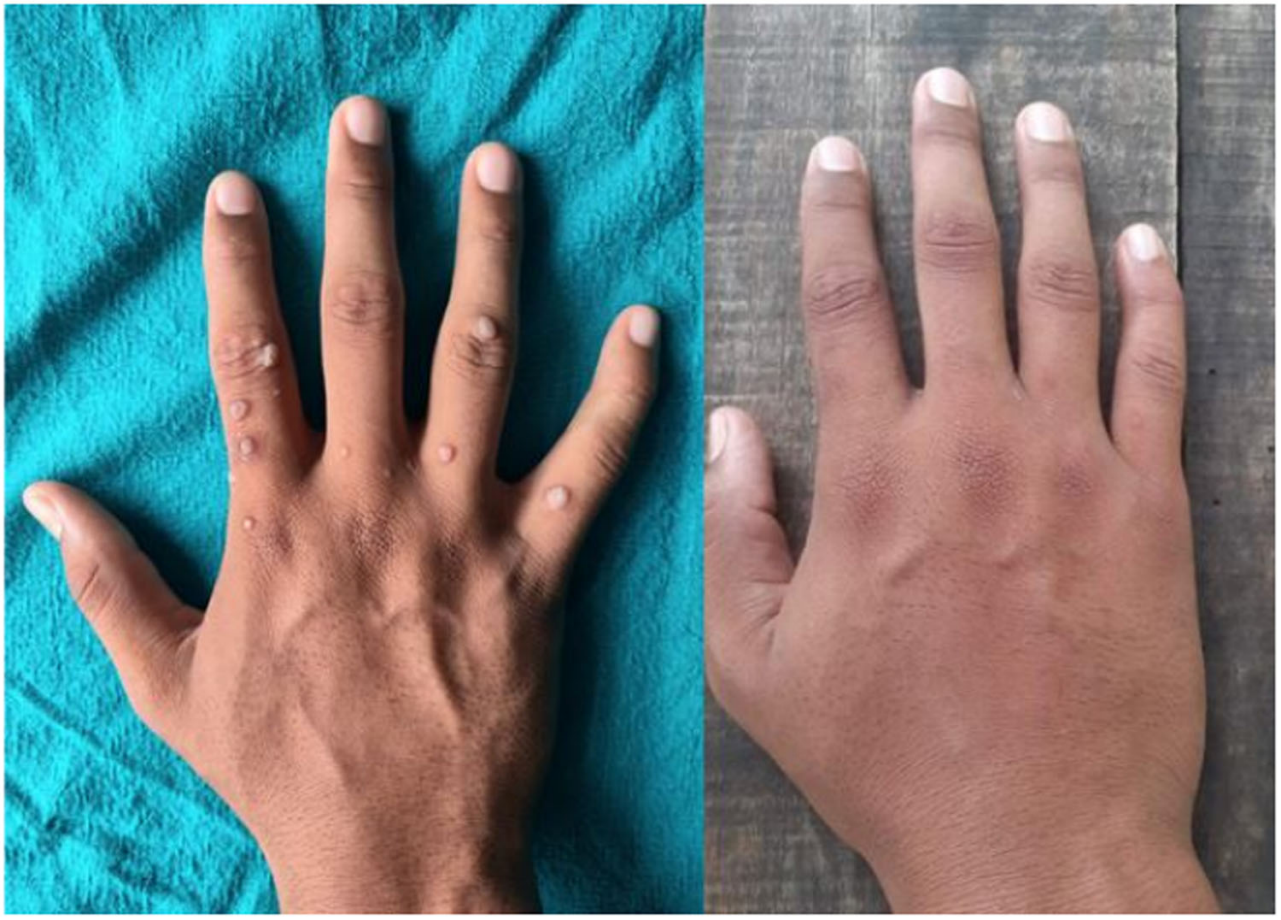
Complete clearance with three sessions of MMR.

**Figure 3:**
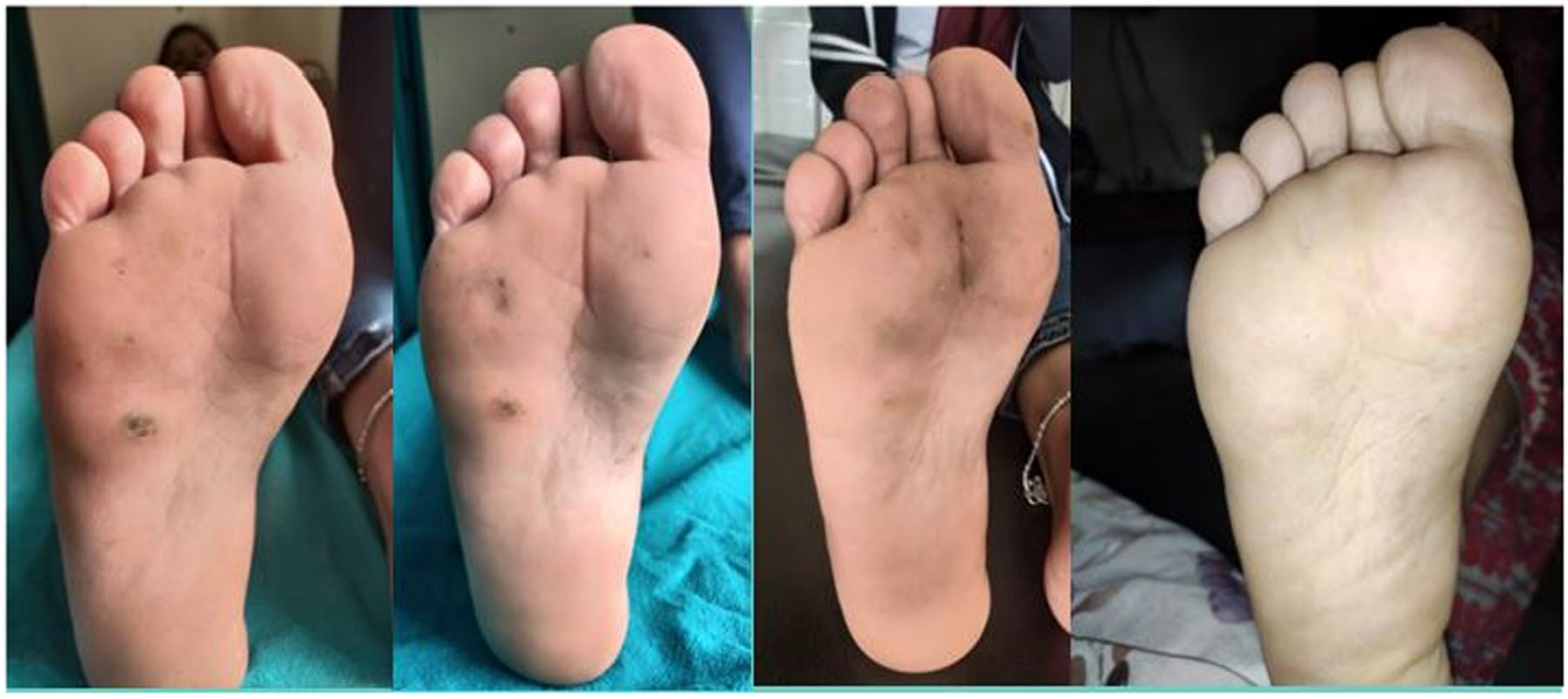
Complete response after four sessions of MMR.

**Figure 4:**
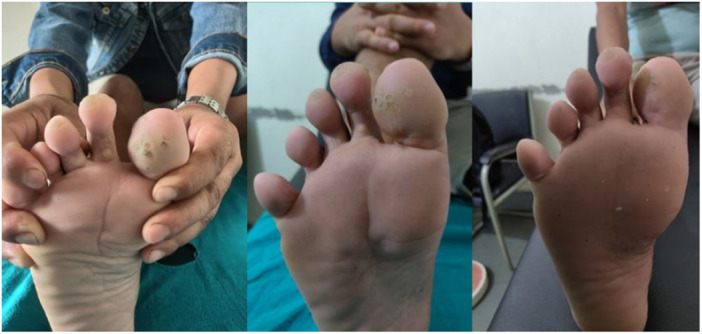
Poor response with five sessions of MMR.

**Figure 5:**
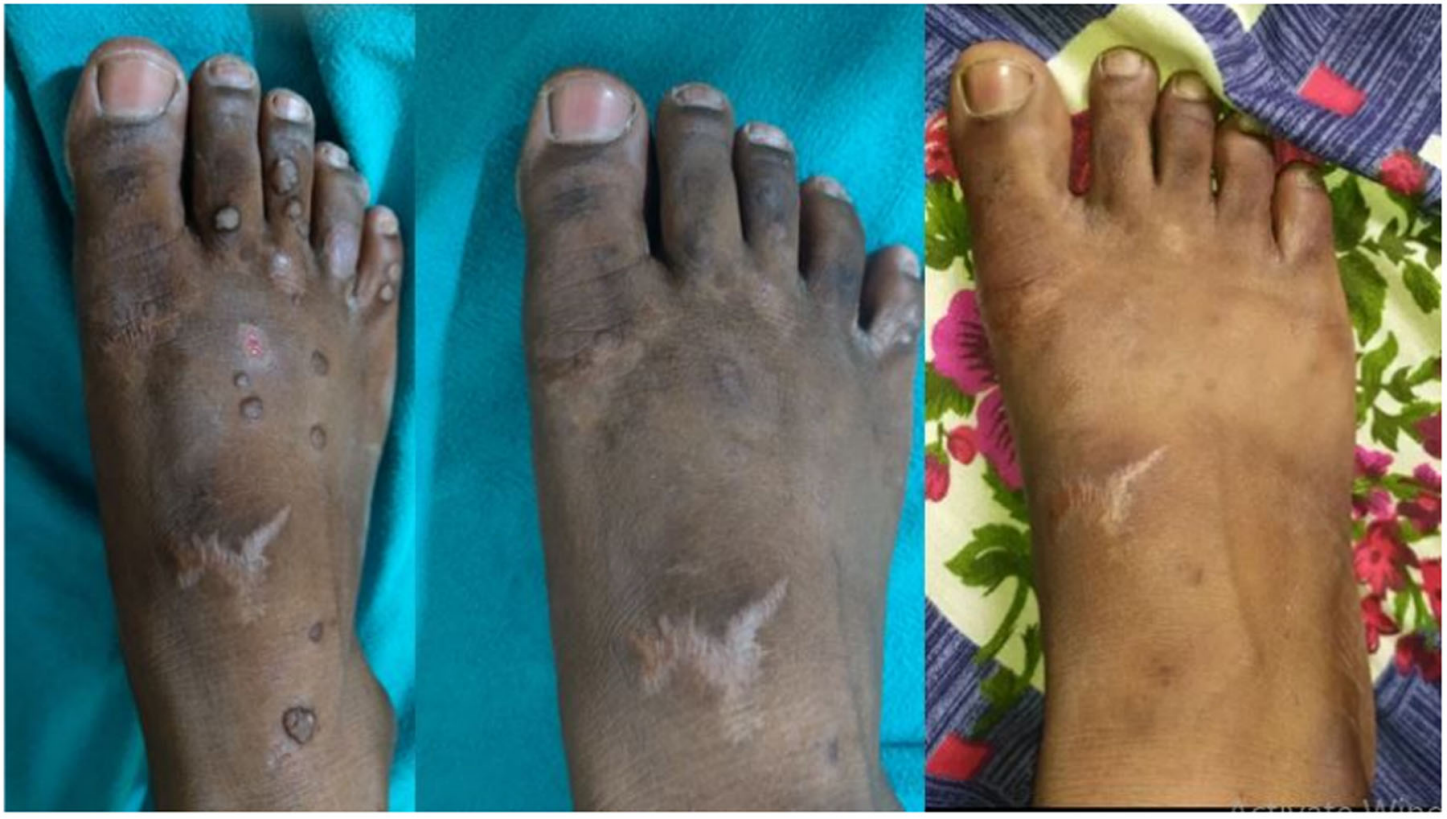
Complete response with three sessions of vitamin D, scar of previous electrocautery is visible.

**Figure 6:**
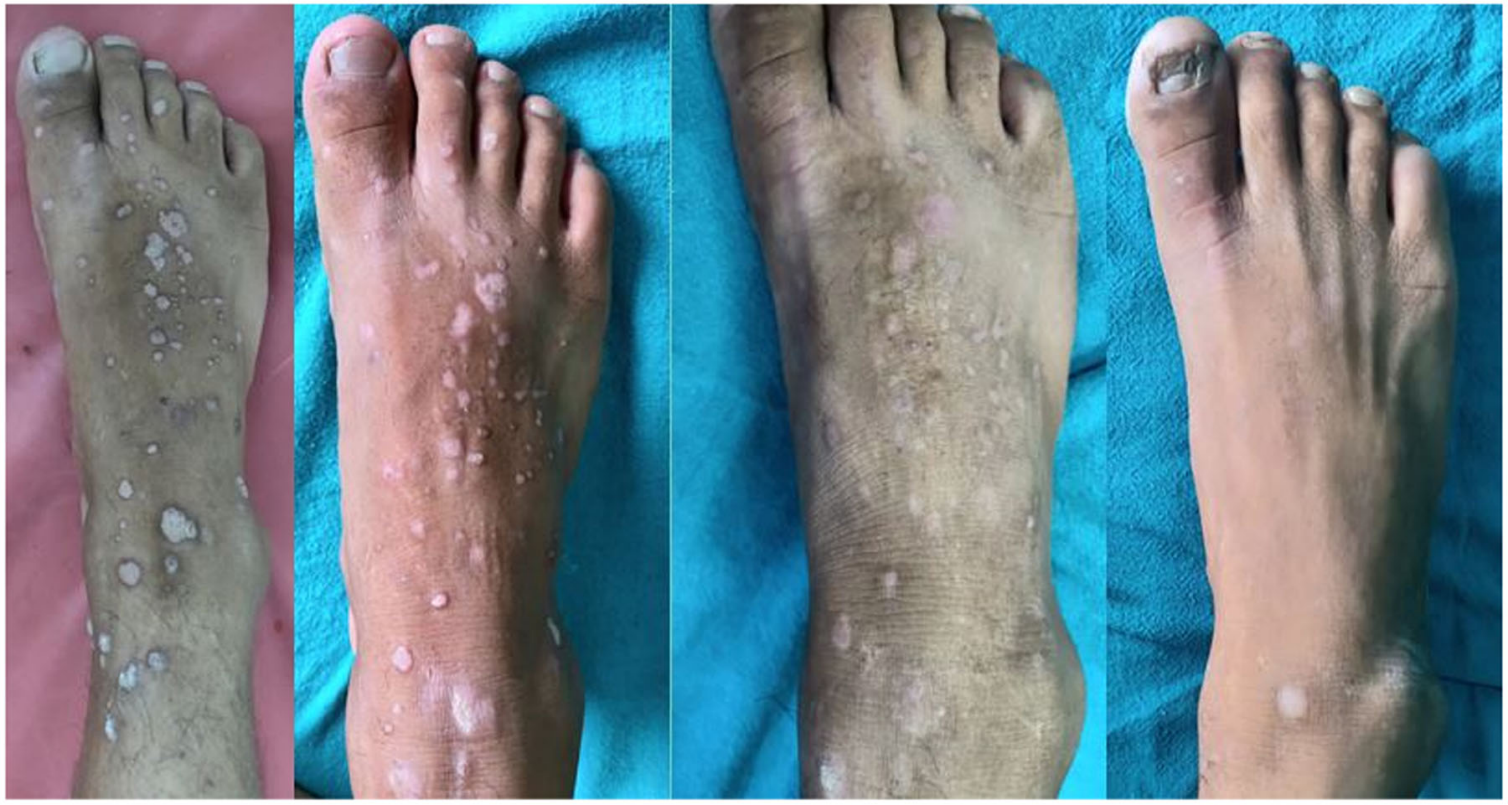
Complete response with five sessions of vitamin D.

**Figure 7:**
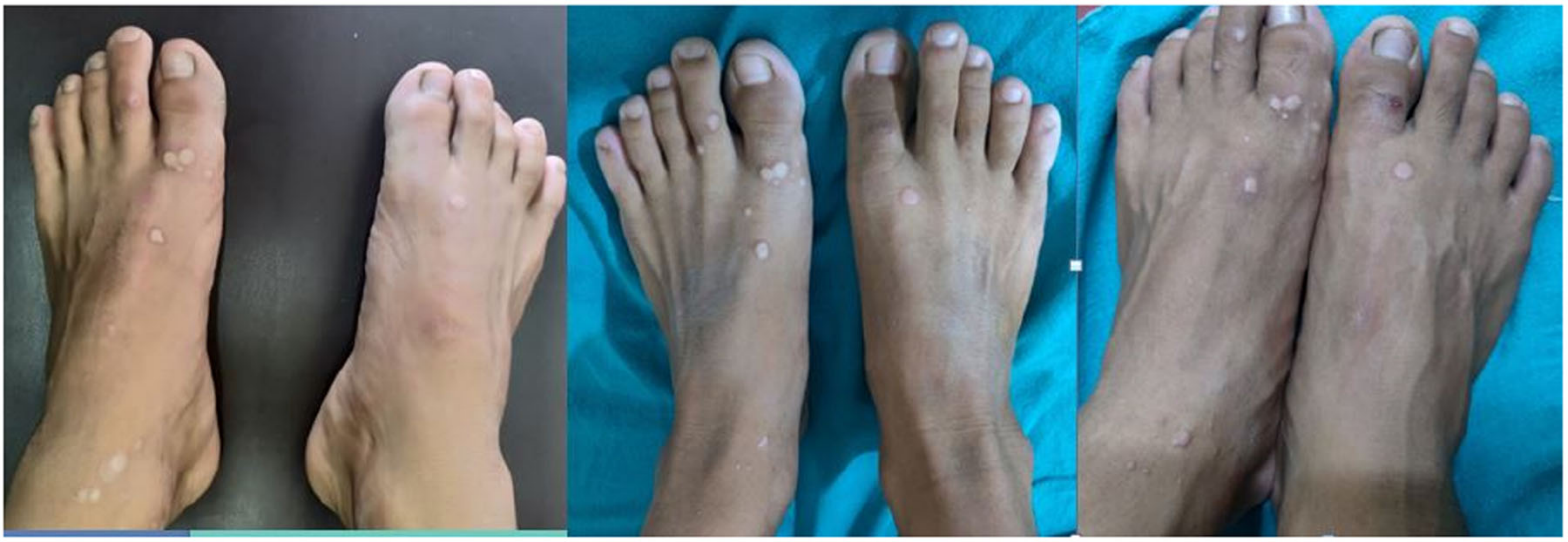
Poor response with even five sessions of vitamin D.

In the MMR group, a complete response was observed in 25 patients (80.6%), an excellent response in 2 patients (6.5%), a good response in 3 patients (9.7%), and a poor or no response in 1 patient (3.2%) (Figures 2, 3, 4). In the Vitamin D3 group, a complete response was seen in 22 patients (73.4%), an excellent response in 6 patients (20.0%), a good response in 1 patient (3.3%), and a poor or no response in 1 patient (3.3%). The difference between the two groups was not statistically significant (*p* = 0.50) (Table 1 and Figures 5, 6, 7).

In the per‐protocol analysis, after excluding patients who did not adhere to the protocol, there were 30 patients in the MMR group and 26 patients in the Vitamin D3 group. In the MMR group, a complete response was observed in 25 patients (83.3%), an excellent response in 2 patients (6.7%), a good response in 2 patients (6.7%), and a poor or no response in 1 patient (3.3%) (Figures 2, 3, 4). In the Vitamin D3 group, a complete response was seen in 20 patients (76.9%), an excellent response in 4 patients (15.4%), a good response in 1 patient (3.8%), and a poor or no response in 1 patient (3.8%). There was no statistically significant difference between the two groups. (*p* = 0.55) (Table 1 and Figures 5, 6, 7).

**Figure 8:**
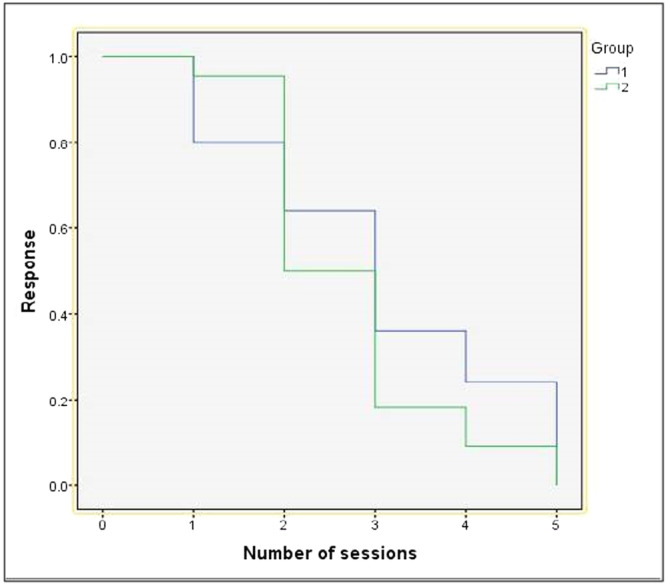
Kaplan–Meier curves comparing the response rate between two groups on each follow‐up visit.

The mean number of treatment sessions required for complete response in the MMR group was 3.04 ± 1.46 while that in the vitamin D3 group was 2.73 ± 1.03. However, the difference was not statistically significant. The Kaplan–Meier curve shows that till two sessions of treatment, vitamin D3 performed better than MMR vaccine but as the number of sessions increased performance of MMR was better than that of vitamin D3. (Figure 8). Only one patient (4.0%) in the MMR group showed recurrence while three patients (13.6%) in the vitamin D3 group showed recurrence in 3 months follow‐up. However, it was not significant statistically (Table 3).

**Table 3:** Comparison of number of sessions required, recurrence rate, and side effects between MMR group and Vitamin D3 group (MMR = Measles, Mumps, Rubella vaccine, *N* = number of participants, OR = odds ratio, CI = confidence interval, R = reference group, NA = not applicable, *significant *p*‐value < 0.05).

Characteristics	Category	MMR group (31) *N* (%)	Vitamin D3 group (30) *N* (%)	Test of significance	*p*‐value	OR (95% CI)
Number of Sessions	1	5 (83.3)	1 (16.7)	*χ* ^2^ = 7.267	0.71	0.60(0.04‐8.730
	2	4 (26.8)	10 (71.4)		0.05*	7.50(1.03‐54.11)
	3	7 (50.0)	7 (50.0)		0.26	3(0.44‐20.31)
	4	3 (60.0)	2 (40.0)		0.57	2(0.18‐22.05)
	5	6 (75.0)	2 (25.0)			R
Recurrence	No	24 (96.0)	19 (86.4)	Fisher's exact test = 1.396	0.33	0.217 (0.023‐2.063)
	Yes	1 (4.0)	3 (13.6)			R
Pain	Yes	31(100)	30 (100.0)	N/A	N/A	N/A
	No	0 (0.0)	0 (0.0)			
Pain duration (days)	≤ 1	26 (83.9)	6 (20.0)	*χ* ^2^ = 25.41	< 0.001*	0.023 (0.002‐0.217)
	2–5	4 (12.9)	14 (46.7)			0.35(0.034‐3.622)
	≥ 5	1 (3.2)	10 (33.3)			R
Pruritus	Yes	1 (3.2)	5 (16.7)	Fisher's exact test = 3.106	0.10	6 (0.657‐54.78)
	No	30 (96.8)	25 (83.3)			R
Swelling	Yes	0 (0.0)	18 (60)	N/A	N/A	N/A
	No	31 (100)	12 (40.0)			
Hyperpigmentation	Yes	1 (3.2)	9 (30.0)	Fisher's exact test = 7.974	0.006*	12.85(1.51‐109.27)
	No	30 (96.8)	21 (70.0)			R
Hypopigmentation	Yes	2 (6.5)	2 (6.7)	Fisher's exact test = 0.001	> 0.99	1.03 (0.136‐7.867)
	No	29 (93.5)	28 (93.3)			R

**Figure 9:**
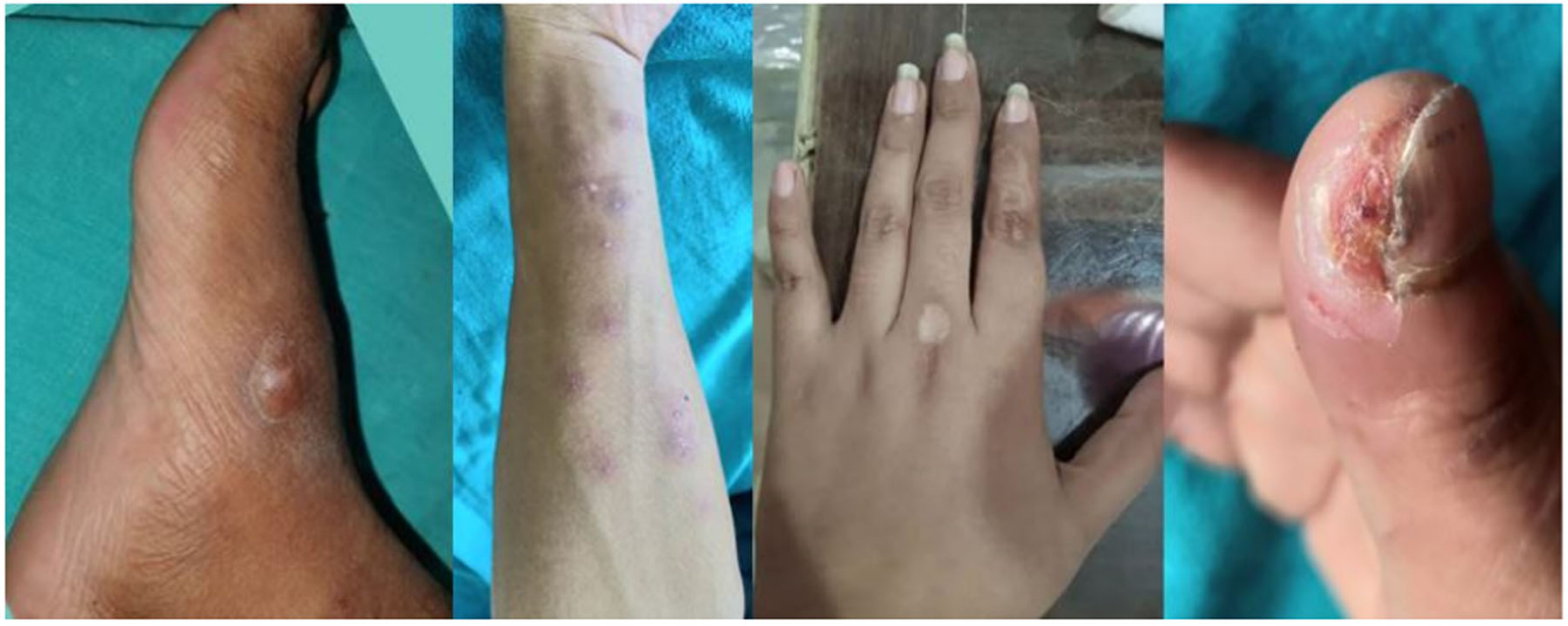
Side effects: Swelling, erythema, hyperpigmentation, hypopigmentation, and ulceration.

The pain was present in all patients of both groups. The median pain duration in the MMR group was 1.0 (IQR: 1.0–1.0), while in the vitamin D group, it was 3.0 (IQR: 2.0–6.5). This variation in duration was statistically significant (*p* < 0.001). Logistic regression analysis showed a significantly longer pain duration in the Vitamin D group compared to the MMR group (*p* < 0.001), indicating a strong association between treatment and pain duration (Table 4). Pruritus was present in a single participant in the MMR group while it was present in 5 (16.7%) vitamin D3 group participants. However, it was not statistically significant (*p* = 0.10). No patients in the MMR group developed swelling. However, 18 (60%) patients in the vitamin D3 group developed swelling with duration ranging from 2 to 110 days, mean duration being 21.67 ± 33.87 days. Hyperpigmentation was present in a single patient in the MMR group and 9 (30.0%) patients in the vitamin D3 group. In bivariate analysis, hyperpigmentation showed a significant association with the outcome (*p* = 0.006), but after adjusting for other variables in the logistic regression model, the association was no longer significant (*p* = 0.597), suggesting that the initial relationship was influenced by confounding factors. Two patients in each group developed hypopigmentation. A single patient in the vitamin D3 group had erythema for 110 days. Two patients in the vitamin D3 group developed ulceration and in both of them, it lasted for 7 days. A single patient in the vitamin D3 group developed fatigue which lasted for 3 days and a single patient in the MMR group developed fever which lasted for 3 days (Figure 9 and Tables 3 and 4).

**Table 4:** Multivariate regression analyses of pain duration, hyperpigmentation, size of largest wart, and the number of sessions (MMR = Measles, Mumps, Rubella vaccine, *N* = number of participants, OR = odds ratio, CI = confidence interval, cm = centimeter, *significant *p*‐value < 0.05).

Characteristics	Category	MMR group (31) *N* (%)	Vitamin D3 group (30) *N* (%)	Crude OR (95% CI)	*p*‐value	Adjusted OR (95% CI)	*p*‐value
Pain duration (days)	≤ 1	26 (83.9)	6(20.0)	0.023 (0.002–0.217)	< 0.001	0.055 (0.011–0.272)	< 0.001*
	2–5	4 (12.9)	14 (46.7)	0.35 (0.034–3.622)	0.38		
	≥ 5	1 (3.2)	10 (33.3)	R			
Hyperpigmentation	Yes	1 (3.2)	9 (30.0)	12.85 (1.51–109.27)	0.006	2.49 (0.084–73.70)	0.60
	No	30 (96.8)	21 (70.0)	R			
Size of largest wart (cm)	0.2–1	14 (45.2)	12 (40.0)	2.57 (0.56–11.72)	0.22	0.989 (0.294–3.325)	0.99
	1–2	8 (25.8)	15 (50.0)	5.62 (1.17–26.85)	0.03		
	≥ 2	9 (29.0)	3 (10.0)	R			
Number of Sessions	1	5 (83.3)	1 (16.7)	0.60 (0.04–8.730	0.71	2.01(0.948– 4.263)	0.07
	2	4 (26.8)	10 (71.4)	7.50 (1.03–54.11)	0.05		
	3	7 (50.0)	7 (50.0)	3 (0.44–20.31)	0.260		
	4	3(60.0)	2 (40.0)	2 (0.18–22.05)	0.571		
	5	6 (75.0)	2 (25.0)	R			

In 9 patients of the vitamin D3 group, vitamin D level was measured after the 3rd dose in 4 patients and after the 5th dose in the remaining 5 patients and it was high in 6 (66.7%) and normal in 3 (33.3%). Among those who developed hypervitaminosis D3, it was seen after the 3rd dose in 3 patients and after the 5th dose in the remaining 3 patients.

## 3 Discussion

Response to MMR vaccine has been variable ranging from 70.4% to 82.4% [13, 19]. In our study, complete response was seen in 25 (80.6%) and in agreement with our results, Shaldoum et al. [20] also reported that among patients receiving MMR, 80% showed complete response and Nofal et al. [15] reported complete response in 81.4%. On the contrary, Na et al. [21] and Saini et al. [22] reported complete response in only 26.5% and 26.44% respectively likely due to differences in study populations, dosing regimens, and wart characteristics.

The clearance rates for IL vitamin D have varied from 40% to 90% in different studies [9, 16] and we observed complete response in 22 (73.4%). Slightly lower response rates were observed by Shaldoum et al. [20] that is, complete clearance in 66.7%. Aktaş et al [23] and Kavya et al. [24] recorded complete clearance in 80% and 78.57% respectively. Although the response rate was higher in the MMR group than the vitamin D3 group it was statistically insignificant. The variation in response rates may be due to differences in the selection criteria of the study population, the sample size, the duration of the study, and the refractory nature of the warts treated. These factors can significantly influence treatment outcomes and efficacy assessments [25].

### 3.1 Treatment Sessions Required for Clearance

The mean number of sessions required for complete clearance in the MMR group was 3.04 ± 1.46, which aligns with Nofal et al. [11] but contrasts with Naseem et al. [26] (2.5 ± 0.57 sessions) and Shaldoum et al. [20] (5.47 ± 0.64 sessions). The discrepancy may stem from differences in the volume of MMR injected per session, as we used 0.5 mL per session compared to 0.3 mL in Shaldoum's study [20].

Contrastingly, in the vitamin D3 group, the mean number of sessions needed was 2.73 ± 1.03. This finding was in agreement with that of Shaldoum et al [20] but was slightly lower than the studies done by Raghukumar et al (3.66) [9], and Kavya et al. (3) [24]. The average number of treatment sessions were insignificantly lower in Vitamin D3 group than MMR group which was consistent with the finding of Shaldoum et al. [20] This suggests that IL vitamin D3 may achieve similar clearance rates in fewer sessions, potentially reducing the burden of repeated interventions for patients.

### 3.2 Recurrence Rates

Only one patient in the MMR group showed recurrence similar to findings of Nofal et al [11] and Na et al [21] while no recurrence was observed by Zamanian et al. [27] In contrast, three patients in the vitamin D group showed recurrence comaparable to Raghukumar et al. (3.33%) [9] and Kavya et al. (2.4%) [24]. Singh et al. [17] and Aktaş et al. [23] observed no recurrence. Although the recurrence rate was higher in the vitamin D3 group, the difference was not statistically significant, indicating comparable long‐term efficacy between the two therapies.

### 3.3 Adverse Effects and Safety Profile

*Pain* was reported in 100% of patients in both groups, aligning with findings from Zamanian et al. [27] (100%), Nofal et al. [15] (85.7%), and Dhope et al. [18] (85%). Abou‐Taleb et al. [28] and Shaldoum et al. [20] also noted significant pain in patients receiving IL Vitamin D, reinforcing that pain is a consistent side effect across studies. However, the duration of pain was significantly different between the two groups, suggesting that while both treatments cause discomfort, the experience may differ in intensity or duration.

*Pruritus* was minimal in the MMR group (3.2%) but more common in the vitamin D3 group (16.7%). This aligns with findings from Nofal et al. [11] (6.1% in the MMR group)and Abou‐Taleb et al. [28] (34.8% in the vitamin D3 group). This support the trend of increased pruritus with IL vitamin D and the duration of itching was also insignificantly higher in the vitamin D group.

*Swelling* was absent in the MMR group but observed in 60% of vitamin D3 patients. El‐. Dhope et al. [18], on the other hand, reported swelling in 20% of patients receiving MMR, potentially due to different injection techniques (e.g., single lesion vs. multiple lesion injections). Among Vitamin D related studies, Kavya et al. [24] also reported high incidence of swelling in 33 (78.57%) patients which resolved without any treatment in 4 weeks while Raghukumar et al [9] reported swelling in only 3.33%, resolving within 1 week. This finding suggests that suggests that localized inflammatory reactions are more frequent with vitamin D3.

*Hyperpigmentation* was significantly higher in the vitamin D3 group (30%) compared to the MMR group (3.2%). Kavya et al. [24] reported hyperpigmentation in only one out of forty‐two patients treated with vitamin D3. This warrants further investigation, as it may impact patient satisfaction.

*Hypopigmentation* was observed in two patients in each group. Unlike other adverse effects, this was not widely reported in previous studies adding a new finding to the literature.

*Erythema* was absent in the MMR group but present in 3.3% of vitamin D3 patients, lasting for an average of 110 days. This is consistent with Raghukumar et al. [9] (5% erythema rate in vitamin D‐treated patients). Dhope et al. [18] and Nofal et al. [11] reported erythema in 4.6% and 25% of MMR‐treated patients, respectively, suggesting that injection‐site reactions are more common with vitamin D3 but not exclusive to it.

*Ulceration* occurred in two vitamin D3‐treated patients, with symptoms resolving within 7 days. This aligns with findings from Singh et al. [17] and El‐Aziz El‐Taweel et al. [16] This finding also suggests higher local inflammation with vitamin D than MMR.

*Systemic side effects* were rare but notable. One patient in the MMR group developed fever, similar to reports from Dhope et al. [18] and Zamanian et al. [27], who described influenza‐like symptoms post‐MMR injection. In the vitamin D3 group, one patient developed fatigue lasting 3 days due to hypervitaminosis D3. In 9 patients of the vitamin D group, vitamin D level was measured and it was high in 6 (66.7%) and normal in 3 (33.3%). Raghukumar et al. [9] and Shaldoum et al. [20] monitored calcium levels in vitamin D‐treated patients, while Kavya et al. [24] and Aktaş et al [23] used clinical assessments for hypervitaminosis D. Importantly, in our study, only one out of six hypervitaminosis D3 cases was symptomatic, suggesting that biochemical monitoring is preferable to relying solely on clinical symptoms.

Strengths of our study include standardized setting and high external validity making study generalizable to similar population. We also have studied the side effects in detail and measured serum vitamin D, which is lacking in other similar studies. On the other hand, single centre study, small sample size, absence of control group, follow‐up of many patients via telemedicine amidst the COVID pandemic, noninclusion of genital warts, unavailability of baseline vitamin D and inability to track vitamin D at regular intervals are the limitations of this study.

In conclusion, this study demonstrates that IL MMR and IL vitamin D3 are both viable immunotherapeutic options for wart treatment. While IL MMR showed a slightly higher clearance rate, IL vitamin D3 required fewer sessions but had a higher incidence and duration of side effects. Further large‐scale, multicenter trials are needed to evaluate the combination of IL immunotherapy with other modalities and to determine the safety profile of IL vitamin D3, particularly in relation to hypervitaminosis D.

## Author Contributions

**Bibisha Baaniya:** conceptualization, data curation, investigation. **Suchana Marahatta:** project administration, supervision, writing – original draft. **Raunak Dahal:** methodology, writing – original draft. **Nidhi Shah:** supervision, validation, writing – review and editing.

## Conflicts of Interest

The authors declare no conflicts of interest.

## Transparency Statement

The lead author Bibisha Baaniya, Bibisha Baaniya affirms that this manuscript is an honest, accurate, and transparent account of the study being reported; that no important aspects of the study have been omitted; and that any discrepancies from the study as planned (and, if relevant, registered) have been explained.

## Data Availability

The data that support the findings of this study are available from the corresponding author upon reasonable request. Due to privacy concerns the data are not publicly available.

## References

[1] M. Kilkenny and R. Marks , “The Descriptive Epidemiology of Warts in the Community,” Australasian Journal of Dermatology 37, no. 2 (1996): 80–86, 10.1111/j.1440-0960.1996.tb01010.x. 8687332

[2] J. C. Sterling , S. Gibbs , S. S. Haque Hussain , et al., “British Association of Dermatologists' Guidelines for the Management of Cutaneous Warts 2014,” British Journal of Dermatology 171, no. 4 (2014): 696–712, 10.1111/bjd.13310. 25273231

[3] L. Luria and G. Cardoza‐Favarato , “Human Papillomavirus.” StatPearls [Internet] (StatPearls Publishing, January 2023). 28846281

[4] L. Leung , “Recalcitrant Nongenital Warts,” Australian Family Physician 40, no. 1–2 (2011): 40–42. 21301692

[5] D. Thappa and M. Chiramel , “Evolving Role of Immunotherapy in the Treatment of Refractory Warts,” Indian Dermatology Online Journal 7, no. 5 (September/October 2016): 364–370, 10.4103/2229-5178.190487. 27730031 PMC5038096

[6] T. D. Horn , S. M. Johnson , R. M. Helm , and P. K. Roberson , “Intralesional Immunotherapy of Warts With Mumps, Candida, and Trichophyton Skin Test Antigens: A Single‐Blinded, Randomized, and Controlled Trial,” Archives of Dermatology 141, no. 5 (May 2005): 589–594, 10.1001/archderm.141.5.589. 15897380

[7] S. Sushantika and N. Singh , “Randomized Comparative Study Assessing the Efficacy of Intralesional Measles, Mumps, and Rubella (MMR) Vaccine and Vitamin D3 in Treating Warts,” Cureus 17, no. 2 (February 2025): e78337, 10.7759/cureus.78337. 40034627 PMC11874879

[8] D. Jakhar , I. Kaur , and R. Misri , “Intralesional Vitamin D3 in Periungual Warts,” Journal of the American Academy of Dermatology 80, no. 5 (2019): e111–e112, 10.1016/j.jaad.2018.11.007. 30447324

[9] S. Raghukumar , B. C. Ravikumar , K. N. Vinay , M. R. Suresh , A. Aggarwal , and D. P. Yashovardhana , “Intralesional Vitamin D3 Injection in the Treatment of Recalcitrant Warts: A Novel Proposition,” Journal of Cutaneous Medicine and Surgery 21, no. 4 (2017): 320–324, 10.1177/1203475417704180. 28384048

[10] S. Kus , T. Ergun , D. Gun , and O. Akin , “Intralesional Tuberculin for Treatment of Refractory Warts,” Journal of the European Academy of Dermatology and Venereology 19, no. 4 (July 2005): 515–516, 10.1111/j.1468-3083.2004.01176.x. 15987315

[11] A. Nofal , E. Nofal , A. Yosef , and H. Nofal , “Treatment of Recalcitrant Warts With Intralesional Measles, Mumps, and Rubella Vaccine: A Promising Approach,” International Journal of Dermatology 54, no. 6 (2015): 667–671, 10.1111/ijd.12480. 25070525

[12] P. A. Achdiat , Yunitasari , H. Usman , and R. H. Maharani , “A Case of Genital and Extragenital Warts Unresponsive to Immunotherapy Using Measles, Mumps, Rubella Vaccine,” International Medical Case Reports Journal 16 (November 2023): 739–746, 10.2147/IMCRJ.S426665. 38020581 PMC10657768

[13] J. Raju , A. V. Swamy , B. L. N. Swamy , and K. R. Raghavendra , “Intralesional Measles, Mumps and Rubella (MMR) Vaccine‐An Effective Therapeutic Tool In the Treatment of Wart,” Journal of Evidence Based Medicine and Healthcare 2, no. 50 (2015): 2349–2562, 10.18410/jebmh/2015/1176.

[14] I. M. A. Kareem , I. M. Ibrahim , S. F. F. Mohammed , and A. A. Ahmed , “Effectiveness of Intralesional Vitamin D 3 Injection in the Treatment of Common Warts: Single‐Blinded Placebo‐Controlled Study,” Dermatologic Therapy 32, no. 3 (2019): e12882, 10.1111/dth.12882. 30920098

[15] A. Nofal and E. Nofal , “Intralesional Immunotherapy of Common Warts: Successful Treatment With Mumps, Measles and Rubella Vaccine,” Journal of the European Academy of Dermatology and Venereology 24, no. 10 (2010): 1166–1170, 10.1111/j.1468-3083.2010.03611.x. 20202055

[16] A. E. A. El‐Taweel , R. M. Salem , and A. H. Allam , “Cigarette Smoking Reduces the Efficacy of Intralesional Vitamin D in the Treatment of Warts,” Dermatologic Therapy 32, no. 2 (March 2019): e12816, 10.1111/dth.12816. 30623542

[17] S. K. Singh , A. Mohan , A. K. Gupta , and A. K. Pandey , “A Comparative Study Between Intralesional PPD and Vitamin D3 in Treatment of Viral Warts,” International Journal of Research in Dermatology 4 (2018): 197–201, 10.18203/issn.2455-4529.IntJResDermatol20180953.

[18] A. Dhope , B. Madke , and A. L. Singh , “Effect of Measles Mumps Rubella Vaccine in Treatment of Common Warts,” Indian Journal of Drugs in Dermatology 3 (2017): 14–19, 10.4103/ijdd.ijdd_1_17.

[19] P. Chauhan , V. Mahajan , K. Mehta , R. Rawat , and V. Sharma , “The Efficacy and Safety of Intralesional Immunotherapy With Measles, Mumps, Rubella Virus Vaccine for the Treatment of Common Warts in Adults,” Indian Dermatology Online Journal 10, no. 1 (January/February 2019): 19–26, 10.4103/idoj.IDOJ_142_18. 30775294 PMC6362737

[20] D. R. Shaldoum , G. F. R. Hassan , E. H. El Maadawy , and G. M. El‐Maghraby , “Comparative Clinical Study of the Efficacy of Intralesional MMR Vaccine vs Intralesional Vitamin D Injection in Treatment of Warts,” Journal of Cosmetic Dermatology 19, no. 8 (2020): 2023–2040, 10.1111/jocd.13272. 31925891

[21] C. H. Na , H. Choi , S. H. Song , M. S. Kim , and B. S. Shin , “Two‐Year Experience of Using the Measles, Mumps and Rubella Vaccine as Intralesional Immunotherapy for Warts,” Clinical and Experimental Dermatology 39, no. 5 (July 2014): 583–589, 10.1111/ced.12369. 24934912

[22] S. Saini , N. Dogra , and D. Dogra , “A Prospective Randomized Open Label Comparative Study of Efficacy and Safety of Intralesional Measles, Mumps, Rubella Vaccine Versus 100% Trichloroacetic Acid Application in the Treatment of Common Warts,” International Journal of Research in Medical Sciences 4 (2016): 1529–1533, 10.18203/2320-6012.ijrms20161223.

[23] H. Aktaş , C. Ergin , B. Demir , and Ö. Ekiz , “Intralesional Vitamin D Injection May Be an Effective Treatment Option for Warts,” Journal of Cutaneous Medicine and Surgery 20, no. 2 (2016): 118–122, 10.1177/1203475415602841. 26294740

[24] M. Kavya , B. Shashikumar , M. Harish , and B. Shweta , “Safety and Efficacy of Intralesional Vitamin D3 in Cutaneous Warts: An Open Uncontrolled Trial,” Journal of Cutaneous and Aesthetic Surgery 10, no. 2 (April/June 2017): 90–94, 10.4103/JCAS.JCAS_82_16. 28852295 PMC5561717

[25] J. C. Fleet , M. DeSmet , R. Johnson , and Y. Li , “Vitamin D and Cancer: A Review of Molecular Mechanisms,” Biochemical Journal 441, no. 1 (January 2012): 61–76, 10.1042/BJ20110744. 22168439 PMC4572477

[26] R. Naseem and S. Aamir , “The Efficacy of Intralesional Measles, Mumps, Rubella (MMR) Antigen in Treatment of Common Warts,” Pakistan Journal of Medical & Health Sciences 7 (2013): 1130–1133.

[27] A. Zamanian , P. Mobasher , and G. Jazi , “Efficacy of Intralesional Injection of Mumps‐Measles‐Rubella Vaccine in Patients With Wart,” Advanced Biomedical Research 3 (2014): 107, 10.4103/2277-9175.129701. 24804181 PMC4009748

[28] D. A. E. Abou‐Taleb , H. A. Abou‐Taleb , O. El‐Badawy , A. O. Ahmed , A. E. Thabiet Hassan , and S. M. Awad , “Intralesional Vitamin D3 Versus Intralesional Purified Protein Derivative in Treatment of Multiple Warts: A Comparative Clinical and Immunological Study,” Dermatologic Therapy 32, no. 5 (September 2019): e13034. 31355514 10.1111/dth.13034

